# Cycling and Spatial Navigation in an Enriched, Immersive 3D Virtual Park Environment: A Feasibility Study in Younger and Older Adults

**DOI:** 10.3389/fnagi.2019.00218

**Published:** 2019-08-16

**Authors:** Ashwin R. Sakhare, Vincent Yang, Joy Stradford, Ivan Tsang, Roshan Ravichandran, Judy Pa

**Affiliations:** ^1^Department of Biomedical Engineering, Viterbi School of Engineering, University of Southern California, Los Angeles, CA, United States; ^2^Department of Neurology, Mark and Mary Stevens Neuroimaging and Informatics Institute, University of Southern California, Los Angeles, CA, United States

**Keywords:** virtual reality, cycling, exercise, younger adults, older adults

## Abstract

**Background:**

Cognitive decline is a significant public health concern in older adults. Identifying new ways to maintain cognitive and brain health throughout the lifespan is of utmost importance. Simultaneous exercise and cognitive engagement has been shown to enhance brain function in animal and human studies. Virtual reality (VR) may be a promising approach for conducting simultaneous exercise and cognitive studies. In this study, we evaluated the feasibility of cycling in a cognitively enriched and immersive spatial navigation VR environment in younger and older adults.

**Methods:**

A total of 20 younger (25.9 ± 3.7 years) and 20 older (63.6 ± 5.6 years) adults participated in this study. Participants completed four trials (2 learning and 2 recall) of cycling while wearing a head-mounted device (HMD) and navigating a VR park environment. Questionnaires were administered to assess adverse effects, mood, presence, and physical exertion levels associated with cycling in the VR environment.

**Results:**

A total of 4 subjects withdrew from the study due to adverse effects, yielding a 90% completion rate. Simulator sickness levels were enhanced in both age groups with exposure to the VR environment but were within an acceptable range. Exposure to the virtual environment was associated with high arousal and low stress levels, suggesting a state of excitement, and most participants reported enjoyment of the spatial navigation task and VR environment. No association was found between physical exertion levels and simulator sickness levels.

**Conclusion:**

This study demonstrates that spatial navigation while cycling is feasible and that older adults report similar experiences to younger adults. VR may be a powerful tool for engaging physical and cognitive activity in older adults with acceptable adverse effects and with reports of enjoyment. Future studies are needed to assess the efficacy of a combined exercise and cognitive VR program as an intervention for promoting healthy brain aging, especially in older adults with increased risk of age-related cognitive decline.

## Introduction

Cognitive decline in older adults is a significant public health issue. However, recent studies have shown that individuals with a lifestyle rich in cognitive and physical stimulation experience less age-related cognitive decline (Barnes and Yaffe, 2011). Exercise and cognitive enrichment are two lifestyle factors that have been associated with a reduced risk of dementia (Barnes and Yaffe, 2011). Exercise enhances hippocampal neurogenesis, synaptic plasticity, and cell proliferation (Van Praag et al., 2005; Erickson et al., 2011; Lautenschlager et al., 2012), while cognitive enrichment promotes the survival of these newly formed cells (Fabel et al., 2009). Recent animal studies have shown that neurogenesis can be enhanced if exercise is combined with cognitive enrichment (Fabel et al., 2009). This has been supported in human studies in which higher cognitive performance was reported after combined physical and cognitive activity compared to either one alone (Lauenroth et al., 2016). Taken together, this suggests that the greatest improvements in cognitive function may be achieved when exercise and cognitive stimulation are performed simultaneously.

Spatial navigation is a key cognitive process that enables daily exploration of the world. Declines in spatial navigation have been shown with age and may result from changes in neural function and structure (Colombo et al., 2017). Virtual reality (VR) has emerged as a promising technology for combined exercise and spatial navigation studies as it provides a safe and controlled environment to monitor physical activity, manipulate experimental parameters, and interact with the user (Zakzanis et al., 2009). Previous studies have shown VR to be an ecologically valid tool for assessing spatial navigation deficits in healthy adults and individuals with neurological disorders, including those with Alzheimer’s disease (Richardson et al., 1999; Cushman et al., 2008; Weniger et al., 2011; Morganti et al., 2013; Cogné et al., 2017). However, only a limited number of studies have utilized VR for exercise, and specifically for cycling (Grealy et al., 1999; Chuang et al., 2003). Only one study, to our knowledge, has combined cycling and spatial navigation in VR, which was conducted in a group of younger adults (Mittelstaedt et al., 2018); thus its application to older adults is unknown. Furthermore, many of these studies have been conducted on non-immersive desktop monitors and projector screens instead of immersive head-mounted (HMD) displays (Grealy et al., 1999; Richardson et al., 1999; Chuang et al., 2003; Cushman et al., 2008; Weniger et al., 2011; Morganti et al., 2013; Cogné et al., 2017).

Recent technological advances have made HMDs an affordable option for immersive VR. HMDs can couple head movement to the position and orientation of the user’s field of view (FOV), creating a sense of presence and engagement in the virtual environment (Shahrbanian et al., 2012). Previous studies have shown presence to be important for performance on spatial navigation tasks in VR (Maguire et al., 1999). However, increased presence with HMDs can often introduce adverse effects, commonly termed simulator sickness in VR, particularly when coupled with locomotion due to incongruence between perceived and actual self-motion (Chance et al., 1998; Sharples et al., 2008; Boletsis, 2017). There is also a concern that older adults are more likely to experience simulator sickness than younger adults due to age-associated deterioration in sensory processing (Kim et al., 2017), possibly exacerbating the severity of sensory conflict present during locomotion in immersive VR.

Thus, immersive HMDs may have higher ecological validity than desktop monitors and projector screens for cycling and spatial navigation in VR, but with the potential for enhanced adverse effects. Moreover, the presence of these adverse effects can significantly impact enjoyment and performance on cycling and spatial navigation tasks in VR. Concerns of enjoyment and performance are enhanced in the older adult population, as older adults typically have less exposure to technology and digital gaming than younger adults. Therefore, the objective of this study was to evaluate the feasibility of cycling and spatial navigation in VR using immersive HMDs in older adults with younger adults serving as a reference group for assessing adverse effects, mood, enjoyment, presence, and performance.

## Materials and Methods

### Participants

A total of 40 adults, including 20 younger adults (25.9 ± 3.7 years old; 21–33 years; and 9 females), and 20 older adults (63.6 ± 5.6 years old; 52–70 years; and 10 females) who were physically capable of cycling participated in this study. All subjects provided written consent to participate in this study, which was approved by the institutional review board and performed in accordance with the 1964 Declaration of Helsinki. Subjects were selected from a convenience sample of local students, staff, and community-dwelling adults. Subjects with known medical conditions contradicting exercise or neurological disorders were excluded from the study.

### Assessments

Performance on the spatial navigation tasks was assessed by total cycling time, mean cycling speed, and percentage of correct decisions in the virtual environment. Self-reported measures of mood, presence, physical exertion, and simulator sickness were collected and described below. All questionnaires have reported good reliability and internal consistency (Kerr and Els Van den Wollenberg, 1997; Witmer and Singer, 1998; Lessiter et al., 2001; Golding, 2006b; Mühlberger et al., 2007).

#### Mood

Mood was assessed using the stress arousal checklist (SAC) (Duckro et al., 1989), which provides a differential measurement of situational stress and arousal. High stress and arousal levels for younger and older adults were defined by cutoff scores of 6.2 and 6.0, and 5.1 and 6.4, respectively, based on normative values for each age group (Wilson and Corlett, 2005).

#### Simulator Sickness

Adverse effects were measured using 3 different questionnaires that assessed pre-post VR simulator sickness, simulator sickness during VR exposure, and historical motion sickness as a child and adult. Simulator sickness after VR exposure was assessed using the simulator sickness questionnaire (SSQ) (Kennedy et al., 1993). The SSQ was also administered before VR exposure to measure baseline levels of pre-existing symptoms including difficulty focusing, headache, eyestrain, and general discomfort. A total sickness cutoff score of 15 based on previous work (Kim et al., 2017) was used to determine if participants experienced notable simulator sickness after VR exposure and to split participants into two adverse effect groups: minimal and notable. This score also represents the 75th percentile of sickness scores reported on a variety of flight simulators, as well as the midpoint for the part of the population that experienced adverse effects when exposed to these flight simulators (Kennedy et al., 1993).

Simulator sickness was also evaluated after each trial using the short symptom checklist (SSC) (Cobb et al., 1999). The SSC is a shortened version of the SSQ containing a subset of 6 symptoms: nausea, eye strain, dizziness with eyes closed, stomach awareness, difficulty focusing, and general discomfort.

Motion sickness was assessed using the motion sickness susceptibility questionnaire (MSSQ) (Golding, 2006a). The global MSSQ was used to evaluate a participant’s susceptibility to motion sickness in nine different modes of transportation (i.e., cars, trains, and ships) as a child (MSSQ-C) and as an adult (MSSQ-A). A table of normative values was used to convert the global MSSQ score to a percentile with higher scores indicating higher susceptibility to motion sickness.

#### Presence

The participant’s subjective experience of presence in the virtual environment was assessed using the ITC sense of presence inventory (ITC-SOPI) (Lessiter et al., 2001). The ITC-SOPI measures presence based on 4 principal factors: spatial presence, engagement, ecological validity, and negative effects. The negative effects factor provides a measure of adverse physiological reactions including dizziness, nausea, headache, and eyestrain.

#### Physical Exertion

Physical exertion levels were assessed using the Borg rate of perceived exertion (RPE) scale (Borg, 1982). The Borg RPE is a graded scale (6 – no exertion, 20 – maximal exertion) that has been shown to correlate highly with heart rate and exercise intensity on a cycle ergometer (Borg, 1982). A peak RPE cutoff score of 12 was used to assess whether younger and older adult participants achieved moderate exercise intensity levels while cycling in the virtual environment (Shigematsu et al., 2004; Scherr et al., 2013).

### Virtual Environment

The virtual environment was developed using Unity 3D (version 2018.1.1f1). Participants viewed the environment through an HTC Vive Pro headset with 110-degree field of view (FOV) and a 90 Hz refresh rate. The environment was run on an Alienware Aurora R7 PC with a core i7-7700 CPU, 16 GB RAM, and a 1080 Ti graphics card.

**FIGURE 1:**
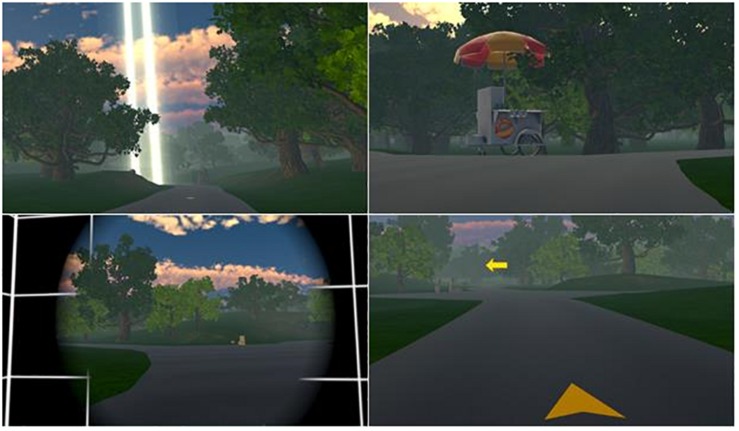
**Top** **left**, beam of light serves as a visual cue to orient the participant in the direction of the destination. **Top** **right**, landmark located at intersection to serve as a visual cue for encoding the correct route in memory. **Bottom** **left**, tunneling effect at intersection to mitigate adverse effects due to sensory conflict when turning. **Bottom** **right**, directional arrows on ground and at intersection guide participant to destination on the first two trials.

The environment consisted of a nature park setting comprised of natural landmarks and animals. Locomotion in the park was achieved by cycling on a custom-built stationary exercise bike, where the handlebar angle and pedal speed were proportional to the movement of a virtual bike. Participants cycled along a network of connected roads with salient landmarks at each intersection to serve as navigational cues. Participants had no avatar to embody but had a stable helmet and nose tip within their FOV. To reduce the likelihood of nausea during locomotion, a technique known as tunneling was employed each time the virtual bike encountered an intersection requiring a turn (Figure 1; Duh et al., 2004; Fernandes and Feiner, 2016; Kemeny et al., 2017). With this technique, the visual field in the periphery of the headset was cropped and replaced with a static black background with white lines, restricting the participant’s FOV, and the amount of optical flow to the periphery of the eye (Duh et al., 2004; Fernandes and Feiner, 2016; Kemeny et al., 2017).

### Experimental Protocol

The experimental protocol consisted of a 1-min practice trial followed by four 2–3 min task trials of cycling in the park, and a set of pre/post assessments. Prior to training in the VR environment, participants stated their current RPE and completed SAC and SSQ questionnaires to establish a baseline of physical exertion, mood, and simulator sickness. Blood pressure (BP) was collected on all older adults. Those with a systolic BP > 170 or diastolic BP > 100 were not allowed to participate in the study following ACSM guidelines for exercise in older adults (Chodzko-Zajko, 2013). Inter-pupillary distance (IPD) was measured for each participant and adjusted accordingly on the HTC Vive headset to increase visual acuity.

#### Practice Trial

Participants sat on the stationary bike while the HMD was placed on their head. Participants then followed the instructions displayed within the game. The first task was to bike around an enclosed oval track for 1 min (30 s in each direction) to adapt to turning and pedaling the stationary bike in the virtual environment.

#### Task Trials

After the practice trial, participants appeared in the park and were instructed to bike to a fountain landmark located approximately 0.5 miles from the start position. This task was completed 4 times under the following trial conditions: learning (2), cued recall, and free recall (Figure 1). In the learning condition, the correct route was identified by yellow arrows located on the surface of the road, as well as a directional blinking arrow at each intersection. The fountain located at the destination was highlighted by a narrow beam of light that vertically spanned the entire FOV. In the cued recall condition, the arrows were removed and only the beam of light remained for navigational guidance. In the free recall condition, no arrows or beam of light were provided, and requiring the participant to rely only on park landmarks for navigation.

At the end of each trial, the participant’s headset was removed and their responses to the RPE scale and SSC were recorded. To optimize engagement and motivation, participants were asked to select a reward after the 1st, 2nd, and 3rd conditions. This included the selection of a basket to go on the virtual bike, an animal companion to ride in the basket, and a song genre to listen to while riding. At the end of the training, participants completed the SAC and SSQ to evaluate mood and simulator sickness and the ITC-SOPI and MSSQ to evaluate presence in the VR environment and general susceptibility to motion sickness.

#### Statistical Analysis

All analysis was performed in SPSS (IBM v24, 2016) (IBM Corp, 2016). The primary outcomes for this analysis were adverse effects, mood, and presence as measured by the SSQ, SAC, and ITC-SOPI, respectively. The SAC and SSQ were measured pre-post VR exposure. An exploratory analysis was also performed to assess physical exertion (Borg RPE), sickness symptoms per trial (SSC), motion sickness susceptibility (MSSQ), and spatial navigation performance. A two-way, repeated measures ANOVA was performed to evaluate group (HY and HO) by time (VR exposure) interactions and group and time main effects on the SAC, SSC, and RPE. The SSC was measured after each of the four trials. The RPE was measured at baseline and after each of the four trials. A one-way ANOVA was performed to evaluate group differences on the ITC-SOPI and MSSQ, as well as performance on the navigation tasks, as these measures were only collected once. A three-way, repeated measures ANOVA was performed to evaluate group, time, or adverse effect level (minimal and notable) differences on the SSC scores reported for each trial. When a significant (*p* < 0.05) interaction was found, *post hoc* comparisons were performed using a paired two-sample *t*-test for time or an independent two-sample *t*-test for group. The Wilcoxon Sign and Mann Whitney *U* tests were used to evaluate the effects of time and group, respectively, on the SSQ. The SSQ was analyzed using non-parametric tests using the change score defined as post-pre, as the SSQ data were not normally distributed. Bonferroni corrections for multiple comparisons were applied based on the number of dependent variables for a given questionnaire.

## Results

Mean, standard deviation, and *p*-values associated with participant demographics and performance in the VR environment are displayed in Table 1. Study outcomes are displayed in Table 2.

**TABLE 1:** Mean, standard deviation, and significance values are reported in this table for participant demographics and performance in the VR environment.

	**All**	**HY**	**HO**	***p*-value**
**Demographics**				
Age (years)	45 ± 19.7	26 ± 3.7	64 ± 5.6	
Gender (M/F)	21/19	11/9	10/10	NS
VR experience (Y/N)	27/13	15/5	12/8	NS
IPD	62 ± 3.3	62 ± 4.3	63 ± 1.7	NS
**Mean blood pressure**				
SBP	141 ± 13.9	–	141 ± 13.9	
DBP	86 ± 11.0	–	86 ± 11.0	
**VR environment**				
Correct decisions (%)	99 ± 2.7	99 ± 1.4	98 ± 3.4	NS
Mean speed (mph)	15 ± 3.9	16 ± 3.5	14 ± 4.3	NS
Trial times (min)				
Total	10 ± 3.0	9 ± 1.7	11 ± 3.9	NS
Mean	2.7 ± 1.0	2.4 ± 0.5	3.0 ± 3.0	0.07
**Mean RPE (score 6 – 20)**				
Baseline	7.7 ± 2.2	8.6 ± 2.8	6.9 ± 0.9	0.77
Trial 1	11.2 ± 2.6	11.9 ± 2.1	10.5 ± 2.8	0.02
Trial 2	11.9 ± 2.5	12.8 ± 2.1	11.2 ± 2.8	0.09
Trial 3	12.5 ± 2.8	13.5 ± 2.2	11.5 ± 3.1	0.01
Trial 4	12.8 ± 3.0	14.1 ± 2.3	11.3 ± 3.1	0.03
**Peak RPE (score 6 – 20)**	14 ± 2.3	12 ± 3.2	13 ± 2.9	0.03

**TABLE 2:** Mean, standard deviation, and significance values are shown for participant responses to presence, mood, and adverse effects questionnaires.

	**All**	**HY**	**HO**	***p*-value**
**Questionnaires**				
MSSQ (percentile)	54 ± 29.9	58 ± 29.0	49 ± 30.9	NS
**Presence (score 1 – 7)**				
Involvement	4.9 ± 0.8	4.8 ± 0.8	4.9 ± 0.8	NS
Sensory fidelity	4.4 ± 1.1	4.6 ± 0.9	4.1 ± 1.2	NS
Adaptation/immersion	5.5 ± 0.7	5.5 ± 0.6	5.5 ± 0.7	NS
Interface quality	3.2 ± 1.4	3.2 ± 1.3	3.2 ± 1.5	NS
**ITC-SOPI (score 1 – 5)**				
Spatial presence	3.4 ± 0.4	3.3 ± 0.4	3.4 ± 0.4	NS
Engagement	3.6 ± 0.5	3.5 ± 0.5	3.6 ± 0.6	NS
Ecological validity	3.6 ± 0.6	3.6 ± 0.6	3.7 ± 0.6	NS
Negative effects	2.4 ± 0.8	2.7 ± 0.9	2.0 ± 0.7	0.04
User enjoyment	3.9 ± 1.0	4.0 ± 1.0	3.8 ± 1.1	NS
**SAC**				
Stress (score 1 – 12.42)				
Pre-exposure	3 ± 2.4	3.7 ± 2.6	2.4 ± 2.2	NS
Post-exposure	2.7 ± 2.4	2.9 ± 2.7	2.5 ± 2.1	
Arousal (score 1 – 9.78)				
Pre-exposure	6.3 ± 2.2	5.4 ± 2.2	7.3 ± 1.9	NS
Post-exposure	6.5 ± 2.2	6.5 ± 2.1	6.5 ± 2.4	
**SSQ**				
Total (score 0 – 235.62)				
Pre-exposure	11 ± 13.8	16 ± 17.2	5 ± 6.4	NS
Post-exposure	25 ± 23.9	30 ± 26.6	19 ± 20.2	
Nausea (score 0 – 200.34)				
Pre-exposure	7 ± 11.3	11 ± 14.3	2 ± 4.2	NS
Post-exposure	25 ± 21.8	30 ± 21.6	21 ± 21.6	
Oculomotor (score 0 – 200.34)				
Pre-exposure	10 ± 13.9	15 ± 16.5	6 ± 8.8	NS
Post-exposure	17 ± 18.1	22 ± 22.3	11 ± 10.6	
Disorientation (score 0 – 200.34)				
Pre-exposure	10 ± 15.8	14 ± 19.7	6 ± 9.6	NS
Post-exposure	23 ± 30.1	26 ± 32.5	20 ± 28.0	
**SSC (score 0 – 18)**				
Trial 1	1.6 ± 1.9	1.9 ± 2.4	1.2 ± 1.4	0.29
Trial 2	1.6 ± 2.0	2.1 ± 2.4	1.1 ± 1.4	0.15
Trial 3	1.6 ± 1.9	1.9 ± 1.8	1.3 ± 2.1	0.42
Trial 4	1.7 ± 2.3	2.5 ± 2.7	0.9 ± 1.6	0.03

**P*-values for questionnaires with pre/post responses denote the significance level associated with the interaction between time (pre/post) and group (young/old).*

### Primary Outcomes of Adverse Effects, Mood, and Presence

#### Simulator Sickness

**FIGURE 2:**
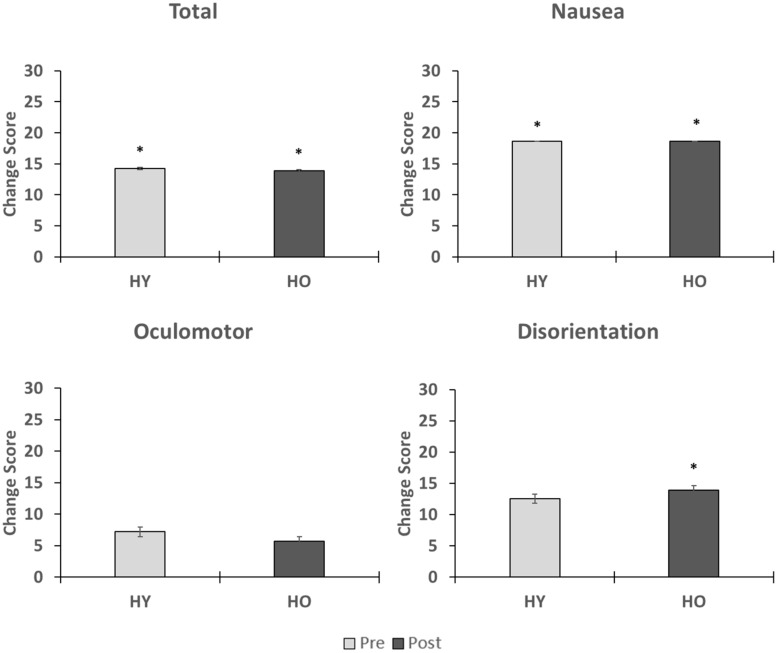
This figure shows the effects of VR exposure on total, nausea, oculomotor, and disorientation levels in the younger and older adults. While symptoms associated each subdomain were enhanced after VR exposure, total sickness levels were less than 15 for both age groups, suggesting that overall adverse effects were minimal. No group differences were observed on the changes scores. ^∗^indicates that a significant difference in symptom severity was found pre-post within an age group.

Simulator sickness as measured by the SSQ (Figure 2) was significantly affected by time (*z* = −3.43, *p* = 0.004, *r* = 0.38), such that symptoms were higher after VR exposure. A total of four adults (1 younger, 3 older, and 10% of study sample) withdrew from the study due to severe symptoms. Analysis of the SSQ subscales showed significant effects of time on symptoms within the subdomains of nausea (*z* = −4.32, *p* < 0.001, *r* = 0.48) and disorientation (*z* = −2.58, *p* = 0.04, *r* = 0.29), such that symptoms were higher after VR exposure (Figure 2). The oculomotor (*z* = −1.92, *p* = 0.06, *r* = 0.21) subdomain was not significantly affected by time. Total sickness (*z* = 0.00, *p* = 1.00, *r* = 0.00), nausea (*z* = −0.07, *p* = 0.95, *r* = 0.00), oculomotor (*z* = −0.14, *p* = 0.89, *r* = 0.02), and disorientation (*z* = −0.48, *p* = 0.64, *r* = 0.05) change scores were not significantly affected by group. Overall, we found that symptoms associated with simulator sickness were enhanced after exposure to the virtual environment. However, total sickness levels were below the cutoff score of 15 for both age groups, suggesting overall adverse effects were acceptable.

#### Mood

**FIGURE 3:**
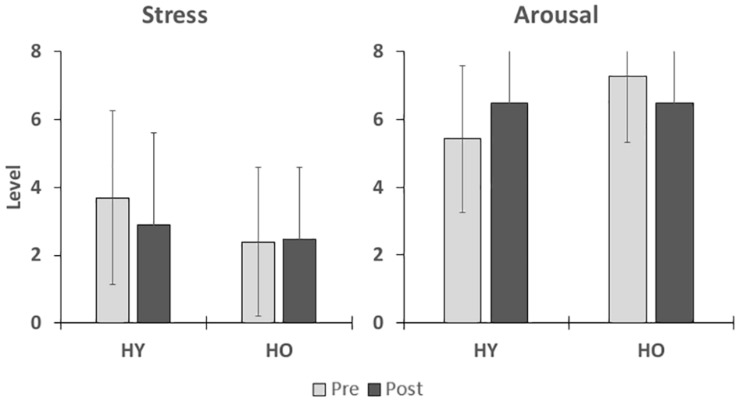
This figure shows the effects of VR exposure on stress and arousal states in participants. No significant differences were found for group or time.

Stress levels as measured by the SAC (Figure 3) were not significantly affected by time [*F*(1,38) = 0.34, *p* = 0.57, η2p = 0.01] or group [*F*(1,38) = 2.74, *p* = 0.11, η2p = 0.07], and there was no interaction between time and group [*F*(1,38) = 2.88, *p* = 0.47, η2p = 0.01]. Arousal levels (Figure 3) were also not significantly affected by time [*F*(1,38) = 0.10, *p* = 0.75, η2p = 0.00] or group [*F*(1,38) = 2.84, *p* = 0.10, η2p = 0.07], and there was no significant interaction between time and group [*F*(1,38) = 5.05, *p* = 0.06, η2p = 0.12]. Mean arousal levels for both age groups were higher than their respective cutoff scores (HY–6.0, HO–6.4) prior to and after VR exposure (Table 2). Mean stress levels for both age groups were also lower than their respective cutoff scores (HY–6.2, HO–5.1) prior to and after VR exposure. Therefore, exposure to the virtual environment did not negatively affect pre-existing high arousal and low stress levels, indicating a state of excitement (Kerr and Els Van den Wollenberg, 1997). On a subjective measurement of pleasure, participants rated the statement “I enjoyed myself.” on a 5-point scale of “strongly disagree” to “strongly agree.” Mean enjoyment levels (Table 2) for the younger and older adults were 4.0 ± 0.9 and 3.8 ± 1.1, respectively, suggesting that participants enjoyed the VR experience.

#### Presence

**FIGURE 4:**
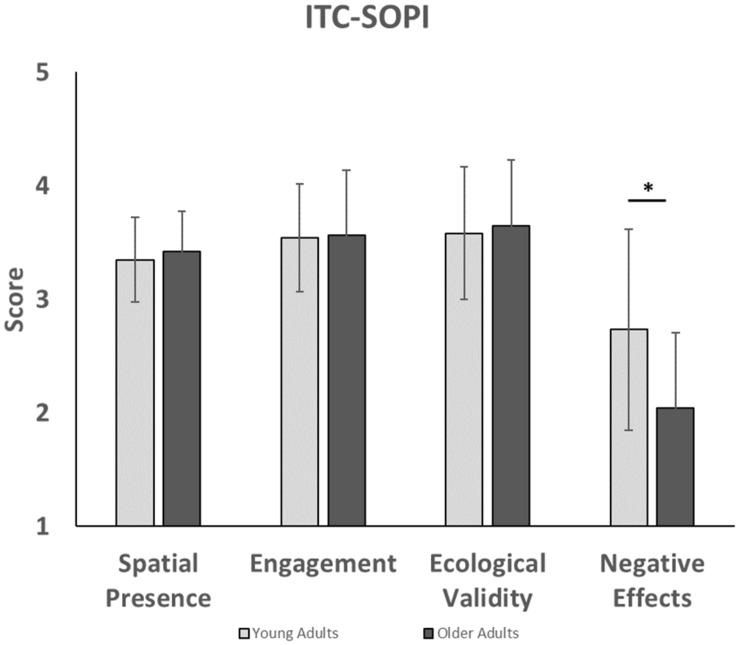
This figure compares presence factors in younger and older adults for the ITC-SOPI questionnaire. Younger adults experienced higher levels of negative effects than older adults. ^∗^indicates that a significant difference was found between younger and older adults with negative effects.

Analysis of the ITC-SOPI (Figure 4) showed that group differences did not significantly affect spatial presence [*F*(1,38) = 0.37, *p* = 0.55, η2p = 0.01], engagement [*F*(1,38) = 0.02, *p* = 0.89, η2p = 0.00], or ecological validity [*F*(1,38) = 0.14, *p* = 0.28, η2p = 0.00]. However, negative effects scores were significantly affected by group [*F*(1,38) = 7.84, *p* = 0.04, η2p = 0.17], such that younger adults had higher negative effects than older adults. Taken together, this suggests that sense of presence in the virtual environment was not significantly different between younger and older adults.

### Secondary Analysis of Physical Exertion, Sickness Symptoms, and Motion Sickness

#### Physical Activity

**FIGURE 5:**
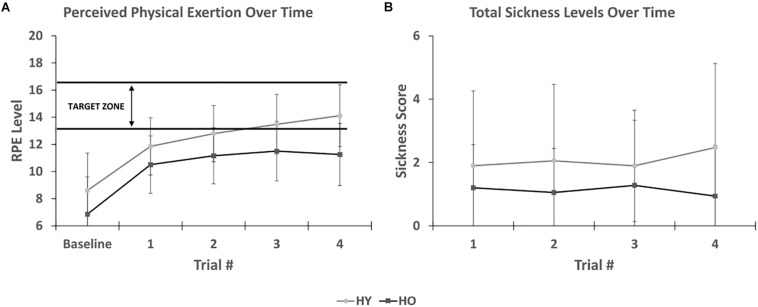
**(A)** Shows that perceived physical exertion levels increases appropriately with time spent in the virtual environment. Younger adults were within the target exercise zone while older adults were approaching it. **(B)** Shows SSC total sickness levels after each trial. No association was found between duration of exposure and symptom severity.

Rate of perceived exertion levels (Figure 5) were significantly affected by time [*F*(1,69) = 95.84, *p* < 0.001, η2p = 0.74] and group [*F*(1,33) = 7.02, *p* = 0.01, η2p = 0.18], such that younger adults reported significantly higher RPE levels than older adults. There was no interaction between time and group [*F*(1,69) = 1.37, *p* = 0.26, η2p = 0.04]. *Post hoc* analysis for time showed that RPE levels increased with each successive trial, suggesting that the participants appropriately perceived an increase in physical exertion with time spent pedaling in the virtual environment. Pairwise comparisons showed significant RPE level differences across all trials (*p* < 0.001), except for the third and fourth trial (*p* = 0.10). *Post hoc* analysis for group showed that mean RPE levels were higher for younger adults (12.2 ± 0.53) than older adults (10.1 ± 0.6). However, peak RPE levels were lower for younger adults (12 ± 3.2) than older adults (13 ± 2.9). A sub-analysis on the relationship between simulator sickness and peak RPE levels found that RPE levels were not significantly associated with SSQ total sickness levels [*F*(1,37) = 0.12, *p* = 0.73, η2p = 0.00], suggesting that physical exertion does not enhance symptoms of simulator sickness in the virtual environment.

#### Sickness Symptoms

The short symptom checklist (Figure 5) showed that total sickness levels were affected by group [*F*(1,33) = 4.92, *p* = 0.04, η2p = 0.13], such that younger adults (1.9 ± 0.4) reported higher scores than older adults (0.8 ± 0.4). There was no interaction between time and group [*F*(1,81) = 1.12, *p* = 0.34, η2p = 0.03]. Total sickness levels were not significantly affected by time [*F*(1,81) = 2.09, *p* = 0.14, η2p = 0.06]. While total sickness levels were not assessed at baseline, this finding suggests that symptom severity only increased during the 1st trial and not with additional time spent in the virtual environment.

In a separate sub-analysis, participants were categorized into two adverse effect groups, minimal or notable, based on an SSQ total sickness cutoff score of 15. From this analysis, we found SSC total sickness levels were significantly affected by adverse effect group for trial 1 [*F*(1,36) = 13.1, *p* = 0.001, η2p = 0.27], trial 2 [*F*(1,34) = 19.25, *p* < 0.001, η2p = 0.36], trial 3 [*F*(1,33) = 20.64, *p* < 0.001, η2p = 0.39], and trial 4 [*F*(1,31) = 12.02, *p* = 0.002, η2p = 0.28]. The mean scores for the minimal and notable adverse effect groups for each trial are as follows: trial 1 (0.8 ± 0.3 and 2.9 ± 0.4), trial 2 (0.7 ± 0.3 and 3.3 ± 0.5), trial 3 (0.8 ± 0.3 and 3.4 ± 0.5), and trial 4 (0.9 ± 0.4 and 3.5 ± 0.6). This suggests that participants with notable adverse effects experienced an onset of symptoms prior to the end of trial 1.

#### History of Motion Sickness

Motion sickness susceptibility (MSSQ) was not significantly affected by group for the MSSQ-C [*F*(1,38) = 0.13, *p* = 0.72, η2p = 0.00], MSSQ-A [*F*(1,38) = 1.18, *p* = 0.28, η2p = 0.03], or global MSSQ scores [*F*(1,38) = 0.44, *p* = 0.59, η2p = 0.02]. In a sub-analysis comparing global MSSQ percentile scores to SSQ total sickness scores, it was found that total sickness levels were positively associated with percentile scores [*F*(1,37) = 5.64, *p* = 0.02, η2p = 0.13], suggesting that participants who are more susceptible to motion sickness are likely to experience higher sickness levels in our VR environment.

#### Spatial Navigation

No significant differences between younger and older adults were found in total cycling times [*F*(1,33) = 3.08, *p* = 0.09, η2p = 0.09], mean cycling speeds [*F*(1,38) = 1.66, *p* = 0.21, η2p = 0.04], or the percentage of correct decisions made while navigating in the virtual environment [*F*(1,38) = 3.54, *p* = 0.07, η2p = 0.09; Table 1].

## Discussion

Recently proposed guidelines suggest a scientific framework for empirically and systematically validating VR therapeutics for health. Consistent with these guidelines, our “VR1” feasibility study is based on patient-reported outcomes for adverse effects, mood, and presence, which is analogous to a traditional Phase 1 clinical trial (Birckhead et al., 2019). Overall, the findings from our study support that cycling and spatial navigation using a HMD display in immersive VR is feasible and enjoyable in both younger and older adults. Younger adults were used as a reference group to ascertain whether significant group differences arise due to the younger adults having more environmental exposure to or feeling more comfortable with technology and digital gaming. The findings of our study suggest that age was not a significant factor in the feasibility of VR in older adults.

Spatial presence, engagement, and ecological validity levels were higher in our study than in similar navigation studies using non-immersive displays (IJsselsteijn et al., 2006). In one such study, participants were seated on a racing bicycle and tasked with cycling along a virtual rural landscape displayed on a wall-mounted projector screen (IJsselsteijn et al., 2006). Participant’s cycled under two conditions: high and low immersion. In the low immersion condition, a moving dot placed on a top-down view of the racetrack was used to represent the participant’s position (IJsselsteijn et al., 2006). In the high immersion condition, a computer-generated cyclist was displayed on the projector screen and controlled by the participants pedaling speed and handlebar rotation (IJsselsteijn et al., 2006). Spatial presence (2.73/1.95), ecological validity (2.98/1.81), and engagement (3.33/2.30) levels reported for both the high and low immersion conditions, respectively, were lower than the values reported in our study. This suggests that immersive HMDs elicit greater psychological involvement, a more natural perception of the environment, and a stronger sense of being physically present in the virtual space than non-immersive displays. HMDs are stereoscopic, providing depth perception for understanding the relative size and position of objects in a virtual environment, which is important for allocentric and egocentric spatial navigation (Bae et al., 2012). Moreover, HMDs provide a high level of fidelity, such that the differences in interactions or experiences between the real world and virtual environment are minimized in comparison to desktop monitors and projector screens (Waller et al., 1998). A high level of fidelity has been shown to enhance the transfer of spatial navigation skills from virtual to real world environments (Waller et al., 1998).

When using immersive VR, simulator sickness is often a concern. Simulator sickness is theorized to be due to postural instability and sensory conflict (Johnson, 2005). Postural instability occurs when an environment or stimuli affects the body’s ability to maintain postural control (Riccio and Thomas, 1991). It is theorized that motion sickness occurs after prolonged maladaptation to the conditions causing postural instability (Riccio and Thomas, 1991). It is also theorized that the severity of motion sickness scales directly with the duration and severity of postural instability (Riccio and Thomas, 1991). Sensory conflict occurs when sensory input to the eyes is incongruent with the vestibular, proprioceptive, and somatosensory systems, causing a mismatch between perceived and expected sensory stimulation in the body (Johnson, 2005). The principal cause of sensory conflict during locomotion is vection (Bonato et al., 2008; Palmisano et al., 2017), defined as visually induced perception of self-motion. Two locomotion techniques used in VR are treadmill walking and cycling and usually involve gait-training (Fung et al., 2006; Mirelman et al., 2011; Kim et al., 2017), and exercise, respectively (Johnson, 2005; Chen et al., 2009; Mestre et al., 2011). Previous studies assessing simulator sickness with treadmill walking have generally reported good tolerability (Kim et al., 2017; Sinitski et al., 2018). To our knowledge, only one study has assessed simulator sickness while cycling in an immersive virtual environment. That study, consisting of healthy younger adult participants, reported a significant increase in adverse effects after cycling on a virtual island (Mittelstaedt et al., 2018).

In our study, younger and older adults also reported an increase in simulator sickness symptoms after cycling in the virtual park, including higher nausea and disorientation levels. However, 90% of our sample successfully completed the study, and total sickness levels for both age groups were within an acceptable range based on cutoff scores established in validation studies on flight simulators (Kennedy et al., 1993). Total sickness levels in our study were also lower than the total sickness levels reported in the virtual island cycling study on younger adults. This can possibly be attributed to the implementation of a stable nose tip and helmet within the FOV, as well as tunneling during turning, which is supported by previous studies that have used these techniques to minimize simulator sickness during locomotion (Duh et al., 2004; Fernandes and Feiner, 2016; Kemeny et al., 2017). We observed a significant group difference on the SSC and negative effects subscale of the ITC-SOPI, such that younger adults reported higher adverse effects than older adults. However, these findings are likely due to younger adults reporting higher sickness symptoms at baseline. Indeed, the pre-post change scores on the SSQ were not significantly different between age groups, suggesting that younger and older adults were similarly affected by VR exposure relative to their baseline symptoms.

In addition to acceptable total sickness levels, we found no association between duration of VR exposure and symptom severity in younger and older adults, suggesting that both age groups acclimated quickly to cycling in the virtual environment. Moreover, our study revealed that exercise did not enhance adverse effects, as we found no association between physical exertion levels and symptoms related to simulator sickness. In fact, peak rates of physical exertion were at high enough intensity levels to be within the recommended range of exercise intensity for health-based and rehabilitative cardiovascular fitness (Shigematsu et al., 2004; Scherr et al., 2013), suggesting that exercising in VR at moderate aerobic intensity levels is tolerable in younger and older adults.

In addition to assessing adverse effects of VR while engaging in physical activity and locomotion, enjoyment is also critical, as approximately 50% of sedentary adults discontinue exercise programs within the first 6 months (Resnick and Spellbring, 2000). Indeed exercise adherence in the older adult population can be a challenge due to lack of motivation, health conditions, and physical discomfort from exercise (Resnick and Spellbring, 2000). In our study, we attempted to enhance enjoyment and motivation by allowing participants to select higher value rewards as more challenging navigation tasks were completed. Prior to the last task, a choice of music was provided, as music has been shown to be the most important factor associated with enjoyment of exercise (Wininger and Pargman, 2003). Our findings support this game design methodology, as ITC-SOPI analysis revealed that most participants enjoyed the virtual experience. Furthermore, according to the SAC, both younger and older adults maintained high levels of arousal and low levels of stress, indicating there was no evidence of an unpleasant experience or a negative shift in hedonic tone that would detract from the overall experience. Taken together, this suggests that younger and older adults enjoyed performing the virtual navigation tasks, even while under increasing levels of physical exertion. This also supports the findings of other studies which have shown higher adherence to exercise when using VR (Annesi and Mazas, 1997), including one in which cycling motivation in VR in older adult cardiopulmonary patients was enhanced and associated with increased cycling times, distance, and total caloric expenditure compared to a non-VR environment (Chuang et al., 2003).

Here we have established the feasibility of cycling and spatial navigation in a virtual environment in both younger and older adults. Both age groups were able to navigate the virtual park environment with 99% accuracy at intersections, with 95% of younger adults, and 75% of older adults able to complete the navigation tasks without error. This suggests that our spatial navigation training paradigm based on cued learning is accessible. Moreover, we have shown that cycling in an enriched virtual environment is enjoyable and tolerable for both age groups, with only 10% of participants discontinuing due to adverse effects. This makes VR a viable tool for interventions that combine exercise and spatial navigation, as well as a safe alternative to cycling in the real world, which requires individuals to continuously maneuver obstacles, such as pedestrians, other bicycles, cars, and environmental barriers. These safety concerns are higher in the older adult population, particularly in individuals with cognitive impairment, as age-associated deteriorations in sensory processing and reaction times can increase the risk of falls and accidents. Moreover, this risk is enhanced when the individual is required to engage in simultaneous cycling and cognitive training, as cognitive resources are now divided between cycling and completing the cognitive task.

However, our study is not without limitations. First, adverse effects in VR may be mediated by gender, as studies have shown that women are more susceptible than men to motion sickness (Koslucher et al., 2015; Munafo et al., 2017). In one such study in which participants utilized a handheld controller to navigate a virtual hallway, it was found that over twice as many women reported motion sickness compared to men (Munafo et al., 2017). Taken together, this suggests that the discontinuation rate may vary from the 10% reported in our study, particularly if the study sample is skewed toward one gender. Second, participants in our study were only exposed to the virtual environment for 10–12 min, which is less time than many exercise interventions that typically last at least 30 min (Anderson-Hanley et al., 2012; Karssemeijer et al., 2019). While we found no association between VR exposure time and adverse effects over a 10–12 min period, sickness levels have been shown to increase with prolonged exposure (Kennedy et al., 2000), and may be enhanced with physical exertion. However, it has also been shown that many individuals can adapt to VR through brief, repeated exposures over time (Kennedy et al., 2000). Therefore, future studies should consider utilizing an adaptation period prior to engaging participants in interventions requiring prolonged VR exposure. Another option is to assess whether the degree of tunneling can be manipulated to make cycling in VR tolerable for individuals that experienced significant adverse effects. However, these studies should also consider that tunneling restricts the user’s FOV, which can reduce immersion and presence, and negatively impact performance on spatial navigation tasks.

One potential tool to screen individuals susceptible to simulator sickness is the MSSQ, which creates a global percentile score based on an individual’s self-reported sickness as a child and adult for nine common modes of transportation. Our study revealed a significant association between global MSSQ motion sickness percentile and total SSQ sickness levels, which has also been reported in other studies (Mittelstaedt et al., 2018). Another option is to measure postural kinematics, as studies have shown that postural instability precedes the onset of motion sickness (Smart Jr et al., 2002). This could be particularly useful for the older adult population, as older adults may already have a baseline level of impaired postural stability due to age-associated deteriorations in the vestibular, proprioceptive, and cognitive systems responsible for maintaining balance (Du Pasquier et al., 2003). Finally, it may be possible to screen participants by assessing their adverse effects after cycling in a virtual environment for 10–12 min, as all affected participants in our study experienced symptoms early within this timeframe.

## Conclusion

Establishing the feasibility of cycling and spatial navigation in immersive virtual environments has clinical importance for both younger and older adults. VR provides clinicians and researchers with a safe and controlled environment for combining spatial navigation with exercise, as well as monitoring cognitive and physical performance. It also provides flexibility to manipulate spatial navigation task difficulty based on one’s fitness level, cognitive status, and age. Moreover, rewards and achievements can easily be incorporated into a virtual environment to enhance enjoyment and increase the likelihood of younger and older adult participation and adherence to an intervention. These benefits make VR a promising tool for interventions aimed at improving cognitive and physical health, especially in older adults at risk for cognitive decline.

## Data Availability

The data collected during this study is available from the corresponding author upon reasonable request.

## Ethics Statement

Human Subject Research: The studies involving human participants were reviewed and approved by the Institutional Review Board (HS-17-00354) at the University of Southern California. The patients and participants provided their written informed consent to participate in this study.

## Author Contributions

AS contributed to the study design and data collection, conducted the data analysis and interpretation, and contributed to the original draft of the manuscript and manuscript revisions. VY and IT contributed to the study design and data interpretation. JS contributed to the data collection and interpretation. RR contributed to the study design. JP conceived the scientific question, contributed to the study design, data interpretation, manuscript writing, and revisions. All authors approved the final manuscript.

## Conflict of Interest Statement

The authors declare that the research was conducted in the absence of any commercial or financial relationships that could be construed as a potential conflict of interest.
